# Changes of Nitric Oxide and Peroxynitrite Serum Levels during Drug Therapy in Patients with Obsessive-Compulsive Disorder

**DOI:** 10.1155/2016/9131680

**Published:** 2016-10-16

**Authors:** Leila Kouti, Mehdi Sayyah, Parisa Mosallanezhad, Sara Kooti, Maryam Aghakoochakzadeh, Kaveh Eslami

**Affiliations:** ^1^School of Pharmacy, Ahvaz Jundishapur University of Medical Sciences, Ahvaz, Iran; ^2^Education Development Center, Ahvaz Jundishapur University of Medical Sciences, Ahvaz, Iran; ^3^Student Research Committee, Ahvaz Jundishapur University of Medical Sciences, Ahvaz, Iran; ^4^School of Pharmacy, Tehran University of Medical Sciences, Tehran, Iran

## Abstract

*Objectives*. Some studies have shown that increased nitric oxide (NO) concentrations may be associated with obsessive-compulsive disorder (OCD). In a few animal researches, enhanced synthesis of NO had reversed the effect of selective serotonin reuptake inhibitors (SSRIs). The present study tries to find the effect of treatment with SSRIs on NO serum levels and its product peroxynitrite.* Patients and Methods*. Patients diagnosed with OCD who are candidates of receiving SSRIs entered this study. Two blood samples were taken from subjects, prior to drug therapy and after the patients had shown some improvements due to their regimen. Serum NO and peroxynitrite levels were measured and their correlation with SSRI use was assessed.* Results*. 31 patients completed this study. Mean concentrations of NO and peroxynitrite prior to drug therapy were 28.63 ± 16.9 and 5.73 ± 2.5 *μ*mol/L, respectively. These values were 18.87 ± 7.55 and 2.15 ± 0.94 *μ*mol/L at the second blood test. With *p* values < 0.05, these differences were considered significant.* Conclusion*. Patients, who showed improvement of OCD symptoms after a mean duration of SSRI monotherapy of 3.531 ± 0.64 months, had lower values of NO and peroxynitrite in their sera compared to their levels prior to therapy. Such results can be helpful in finding a predictive factor of response to therapy and augmentation therapy with future drugs that target NO synthesis.

## 1. Introduction

Nitric oxide (NO) is a free radical, known to lead to neurodegeneration when overproduced in some disorders, such as Parkinson's disease, multiple sclerosis, stroke, and some psychiatric disorders (e.g., schizophrenia, mania, and anxiety) [1, 2].

Some studies have shown that increased NO concentrations may be associated with disorders of obsessive-compulsive spectrum. According to these studies, some oxidants such as NO can act as an inflammatory agent in these disorders [3, 4].

The main neurotransmitter involved in the pathophysiology of obsessive-compulsive disorder (OCD) is serotonin. Thus, selective serotonin reuptake inhibitors (SSRIs) are the first choices of treatment, since they enhance the serotonergic activity. But psychiatrists sometimes have difficulties with drug therapy of OCD, and some patients show resistance to the approved drugs or respond very slowly [5]. Therefore, studies that aim other etiologies and causes of OCD can be helpful in terms of finding new drugs with different mechanisms, targeting other possible causative factors of this disorder.

Some animal studies showed that SSRIs' effects can be reversed by enhanced synthesis of NO, but data in this area is still conflicting [6, 7]. And some found antidepressant and anxiolytic effects of drugs that inhibit NO production (NO synthase inhibitors) [8].

Several studies have explored the values of serum NO in patients with OCD compared with healthy subjects, but our study is conducted to evaluate these levels in patients before and during treatment [9]. Other nitrogen species such as peroxynitrite (byproduct of NO synthesis) measurement can lead to a better prediction of this correlation [2].

We aimed this study at finding the effect of treatment with SSRIs on NO and peroxynitrite serum levels. To our knowledge it is the first study to assess the impact of drug therapy on these levels in patients with OCD.

## 2. Patient and Methods

This study was conducted in a psychiatric outpatient clinic from March to September 2015 by the permission of Ahvaz Jundishapur University of Medical Sciences ethics committee. Otherwise healthy patients diagnosed with OCD, by a board certified psychiatrist, who were candidates of receiving first line drugs of choice for treatment (an SSRI) and naïve to therapy entered this study voluntarily.

Inclusion criteria were age 18–60 years, taking only one drug of the SSRI class, taking no other psychiatric medications, signing the consent form, no contraindication for giving a blood test, not having any disease or state that affects NO production, such as diabetes, rheumatoid arthritis, neurodegenerative disorders, malignancies, chronic kidney disease, other psychiatric disorders, strict vegetarian diet, cigarette smoking, and use of certain drugs (nitrates, antioxidants, insulin, statins, phosphodiesterase inhibitors, pentoxifylline, glucocorticoids, N-acetyl cysteine, selegiline, and coenzyme Q10) [2, 9].

Exclusion criteria consisted of inability to perform the second blood test, noncompliance to the treatment, discontinuation of drug therapy for any reason, other axis I disorders, pregnancy, and development of any condition affecting NO production.

The prescribed SSRI for each patient was individually selected by their psychiatrist, according to their specific state. No other treatment methods (e.g., cognitive behavioral therapy) were used for patients.

Two blood samples (3 cc each) were taken from subjects, one at the time of the diagnosis and the other after the patients showed some improvements in their symptoms (but still needed to continue with their medications since their drug course was not over).

The blood samples were centrifuged at 3000 rpm for 3 minutes and the patients' sera were separated and kept at −80 degrees centigrade until the time of assay.

For measuring NO we used nitrate/nitrite colorimetric assay kit (Cayman®, Ann Arbor, MI, USA). This kit measures NO by Griess method which is a very well recognized technique to assay nitrate/nitrite [10, 11]. The absorbance was read by an ELISA plate reader (Synergy, BioTek®, Winooski, VT, USA) at 540 nm. NO levels were then calculated using the standard nitrate absorbance concentration curve provided by the manufacturer [2, 12].

Peroxynitrite serum level was measured by hydroxyl (OH) radical and peroxynitrite (ONOO) detection kit (Cell Technology, Mountain View, CA, USA). The fluorescence activity was assessed by ELISA plate reader (Synergy, BioTek, Winooski, VT, USA) at an excitation of 488 nm and emission of 515 nm [2].

### 2.1. Statistical Analysis

The results were described as mean ± standard deviation (SD) for quantitative variables. Frequencies were shown by percentages for qualitative variables. Paired *t*-test and generalized estimating equations were used to analyze the data. Results of duration of disease were analyzed by Student's *t*-test. A *p* value of <0.05 was considered significant.

## 3. Results

Thirty-two patients entered this study, one of them dropped out of the study due to pregnancy. 46.7% were female and 53.3% male and aged 36.2593 ± 11.40 years. The mean ± SD for duration of disease and duration of therapy were 10.40 ± 4.98 months and 3.531 ± 0.64 months, respectively (some patients had been suffering from OCD symptoms for a long period before visiting a psychiatrist).

Table 1 shows the concentrations of NO and peroxynitrite serum levels before and during drug therapy. With a *p* value less than 0.05, there is a significant difference between these values. We found no significant correlation between gender and NO levels (*p* value = 0.0522), nor with the duration of the therapy (*p* value = 0.529) and duration of disease (*p* value = 0.527). But there was a significant relationship between NO serum levels and age (*p* value = 0.001). Patients of older age had significantly higher NO and peroxynitrite levels.

The prescribed SSRIs for our patients were Zoloft (30%), paroxetine (22%), fluoxetine (19%), citalopram (11%), sertraline (12%), and fluvoxamine (6%).

Peroxynitrite serum levels at the time of diagnosis were 5.73 ± 2.5 *μ*mol/L. After taking SSRI drugs, this value changed to 2.15 ± 0.94 *μ*mol/L in our patients. With a *p* value less than 0.05, this difference was considered statistically significant.

## 4. Discussion

Our results show that, with a mean duration of SSRI monotherapy of 3.531 ± 0.64 months, the serum levels of NO and peroxynitrite reduced significantly in patients with OCD. In other words, patients who showed improvements of OCD symptoms had lower values of NO and peroxynitrite in their sera. In other studies comparing the NO levels of patients with OCD with healthy controls, elevated levels were seen in patients as well [13].

There are studies evaluating the impact of SSRI therapy on NO levels in major depression (MD), such as a study by Herken et al. The authors evaluated 36 patients with MD before and after 8 weeks of drug therapy. Significantly lower NO serum levels were seen in patients after 8 weeks [14]. In another previous study by Selek et al., 30 patients with bipolar disorder showed significant reduction of NO levels after 30 days of hospitalization [15].

It seems that similar results can be obtained with treatment of patients with OCD. This also shows that one of many natures of OCD can be inflammation, and oxidative stress can have a role in progression of this disorder. In other neurodegenerative disorders, as the patient's situation worsens and the severity increases, the serum NO levels will be increased [2, 16]. In a study by Minutolo and colleagues, NO levels of patients with schizophrenia were higher in patients with more severe disorder and lowered with drug therapy with antipsychotics [17].

Atmaca et al. determined the NO values of 23 patients diagnosed with OCD and naïve to therapy and compared their levels to healthy controls. The mean concentration of NO was 39.4 ± 12.8 *μ*mol/L (our similar patients had a mean value of 28.63 ± 18.87 *μ*mol/L prior to therapy). The NO level was measured by Griess reaction as well [9]. We did not compare different SSRIs' effect on NO levels, since the number of patients treated with each drug varied significantly.

Our data indicated that when patients with OCD respond to SSRI therapy, their NO and peroxynitrite levels will reduce. This reduction's relationship with OCD shows a possible inflammatory nature of this disorder and the role of NO production in its symptom.

Although in our study we introduced NO as a toxic agent with a role in the pathogenesis of OCD, nitric oxide can be beneficial in many biological events (it regulates blood flow, including cerebral blood flow, is a neuromessenger, and kills tumors). Its production in neurons does not necessarily cause toxicity and cell death. Nitric oxide clearance from brain to blood is rapid and has a very short half-life [18, 19]. Peroxynitrite, on the other hand, is a neuroinflammatory agent. It forms when superoxide reacts with NO. There are evidences that NO's toxicity in cells is due to peroxynitrite formation rather than its direct effect [20]. Meanwhile, Pacher and colleagues noted that a 10-fold increased rate of NO production will cause 100-fold peroxynitrite level rise [18]. Therefore in the present study we assessed both of these values in order to have a better evaluation of the results and NO's contrasting roles.

## 5. Conclusion

The results of the present study show that when patients with OCD and naïve to therapy are treated with SSRIs, their serum NO and peroxynitrite levels can change. When they show signs of improvement due to this drug therapy, these levels are lower than their baseline.

Our study is significant in that it evaluated the possible existing relationship between the response to drug therapy and a serum factor (NO and peroxynitrite). Such results can be helpful in finding a predictive factor of response to therapy in addition to using augmentation therapy with future drugs that target NO synthesis [21]. Due to the complex nature of OCD, other oxidants such as peroxynitrite assessment can help us have a more precise prediction of SSRI effect on NO production [22, 23].

## Figures and Tables

**Table 1:** Correlation of NO and peroxynitrite serum levels with drug therapy.

Patients (*n* = 31)	Prior to drug therapy	After improvement with drug therapy	*p* value
Nitrate levels (mean ± SD)	28.63 ± 18.87 *µ*mol/L	16.98 ± 7.55 *µ*mol/L	0.048
Peroxynitrite levels (mean ± SD)	5.73 ± 2.5 *µ*mol/L	2.15 ± 0.94 *µ*mol/L	0.020
